# Supplementary material for: Use of Traditional Mongolian Medicine in Children with Concussion
Source: Medicines (Basel). 2022 Dec 30;10(1):5. doi: 10.3390/medicines10010005 (PMC9863045; doi:10.3390/medicines10010005)
Supplement: Supplementary file 1 [file medicines-10-00005-s001.zip › medicines-2076496-supplementary.pdf]

medicines

Supplementary material. Questionnaire (Translated from Mongolian)

Chapter 1. About the child's guardian

1. Age
2. Gender of guardian (parents or grandparents)
   - o Male
   - o Female
3. Your education level
   - o Primary school
   - o Lower secondary
   - o Upper secondary
   - o Higher education (College)
   - o Higher education (University)
   - o Masters, PhD
4. Your current proposition
   - o Civil servant
   - o Private sector worker
   - o Self-employed
   - o Farmer
   - o Unemployed
   - o Group / paramedical
   - o Student
   - o Retiree
5. Religion
   - o Buddhism
   - o Christian
   - o Islamic
   - o No religion
   - o Others
6. Your child's age
   - o 0-6 months
   - o 6-12 months
   - o 12-24 months
   - o 2-3 years old
   - o 3-4 years old
   - o 4-5 years old
   - o 5-6 years old
   - o 6-7 years old
   - o 7-8 years old
   - o 8-9 years old
   - o 9-10 years old
7. The gender of your child
   - o Male
   - o Female
8. Has your child ever had a concussion?
   - o Yes
   - o No
   - o Unknown

Chapter 2. About concussions that have happened to your child.

1. How many times has your child hit his head?
2. What are the (most common) causes of concussions in your child?
   - o Fall from height (Fall from bed/chair/table/lap)
   - o Hit the head with something
   - o Tripped and fell on the sidewalk

- Motor vehicle accidents
- Assault/Intentional damage
- Sports injuries

3. Has your child had an intracranial (brain contusion or hemorrhage) or skull fracture?
   - Yes
   - No
   - Unknown
4. In which part of the head did your child suffer an injury?
   - Forehead
   - Parietal bone
   - Sphenoid bones
   - Occiput
   - Others
5. Has your child lost consciousness after a head injury?
   - Yes
   - No
   - Unknown
6. What physical symptoms did your child experience immediately after the concussion?
   - Headache
   - Vomit
   - Tired
   - Fatigue
   - Cry
   - Diarrhea
   - Head fever
   - Appetite down
   - Balance problem
   - Dizziness
   - Visual problems
   - Sensitivity to light
   - Sensitivity to noise
   - Numbness / Tinging
   - Hearing loss
7. Has your child experienced any cognitive changes since the concussion?
   - Feeling mentally foggy
   - Difficulty concentrating
   - Difficulty remembering
   - Memory loss
   - Decreased concentration
   - Tired
   - Poor sleep
   - Undetected
8. What emotional changes did your child experience immediately after the concussion?
   - Crying
   - Angry
   - Sadness
   - Emotions are unstable
   - Increase in emotions
   - Undetected
9. What steps do you take after a child's concussion (usually)?
   - Visit a Bariachi
   - Visit a Traditional Medical hospital or a doctor
   - Visit a western medical hospital or a doctor
   - Observed without treatment

10. Please answer why you make the above choice (if you choose a bariachi)?
    - By your own beliefs
    - As suggested by others
    - Through previous and others' experiences
11. Was there a need to go to the bariachi after making other choices without going to the bariachi?
    - Yes
    - No
12. How many days does it usually take to be treated with massage therapy?
    - 1 day
    - 1-3 days
    - 1-3 days
    - 3-5 days
    - 5-7 days
    - None
